# Integrating a Parenting Assessment into Practice: Pediatric Providers’ Time and Perspectives

**DOI:** 10.1007/s10995-024-03984-6

**Published:** 2024-09-16

**Authors:** Amber J Cooke, Tahra I Attar, Victoria L Carr, Anna C Whitney, Rory J Tinker, Kathryn L Carlson, Merrill M Stoppelbein, Laura A Jana, Seth J Scholer

**Affiliations:** 1 Vanderbilt Department of Pediatrics, Monroe Carell Jr. Children’s Hospital at Vanderbilt, Doctors’ Office Towers, 8th Floor, 2200 Children’s Way, Nashville, TN 37232 USA; 2 Penn State’s Edna Bennett Pierce Prevention Research Center, College of Health and Human Development, Pennsylvania State University, 314 Biobehavioral Health Building, University Park, PA 16802 USA

**Keywords:** Parenting, Child abuse prevention, Adverse childhood experiences, Pediatric primary care

## Abstract

**Purpose:**

To integrate a parenting assessment into primary care and assess pediatric providers’ time needed to review it and their perceptions of the process.

**Description:**

The Quick Parenting Assessment (QPA) is a validated, 13 item parent support tool that assesses for healthy and unhealthy parenting practices. Higher QPAs indicate more unhealthy parenting being used. In a clinic serving low-income parents, the QPA was integrated into the 15 month, 30 month, 5 year, and 8 year well child visits. After each well child visit in which the QPA was administered, providers were invited to complete a one-page survey—315 surveys were included in the analysis.

**Assessment:**

Most QPAs (78.7%) were low risk (QPA <  = 2), 14.6% were medium risk (QPA = 3–4), and 6.7% were high risk (QPA > 4). The median time was 15–30 s to review low risk QPAs and 30 s to 1 min to review high risk QPAs. For most QPA reviews, health care providers reported that the QPA increased their objectivity in determining the level of support needed (68%), facilitated communication about parenting (77%), and increased the value of the visit (68%).

**Conclusion:**

A validated parenting assessment tool, integrated into pediatric primary care, appears to work for pediatric health care providers. These findings have implications for supporting parents in pediatrics, value-based care, and disease prevention.

**Supplementary Information:**

The online version contains supplementary material available at 10.1007/s10995-024-03984-6.

## Significance

**What is Already Known on this Subject?:**

Parenting assessments, integrated into primary care, could support parents and reduce poor outcomes, but they are not used routinely. The Quick Parenting Assessment (QPA) is a validated 13-item parenting assessment, associated with an increased risk of behavior problems.

**What this Study Adds?:**

A validated parenting assessment tool was integrated into the well visit in a pediatric clinic. Providers reviewed the tool in less than a minute or two and responded positively to the integration process, suggesting a way to mitigate ACEs.

**Supplementary Information:**

The online version contains supplementary material available at 10.1007/s10995-024-03984-6.

## Purpose

There are compelling reasons for pediatric health care providers to consider integrating parenting assessments into pediatric primary care. First and foremost is health. Positive parenting behaviors lay the foundation for health, (National Academies of Sciences, 2016) potentially mitigating adverse childhood experiences (ACEs) which are associated with many poor outcomes including lung disease, heart disease, obesity, alcoholism, illicit drug use, violence, child abuse, suicide, unemployment, and homelessness (“CDC: Adverse Childhood Experiences”, 2020; Anda et al., 2006; Felitti et al., 1998; Herman et al., 1997). Unhealthy parenting practices are linked with ACEs and child abuse (Afifi et al., 2017; Felitti et al., 1998). In the original ACEs study, a participant who reported that, prior to their 18th birthday, their “parent often pushed or grabbed” them, they were categorized as having been physically abused (Felitti et al., 1998). If a participant reported that a “parent often humiliated them,” they were categorized as having been emotionally abused (Felitti et al., 1998). Pushing, grabbing, and humiliation are parenting disciplinary behaviors. Spanking is yet another example of a parenting behavior that is linked to ACEs and child abuse (Afifi et al., 2017). Second, unlike some ACEs such as divorce, incarceration, and community violence, parenting behaviors are modifiable (Gershoff et al., 2017). Third, parents recognize pediatricians as a source for advice about discipline (Taylor et al., 2013). Fourth, if unhealthy parenting is identified, there are proven ways for pediatricians to respond with evidence-based interventions (Jeong et al., 2021; Smith et al., 2020). Finally, to assess parenting, validated parenting assessment tools are available (Hurley et al., 2014) (Sausen et al., 2022).

Despite reasons to do so, parenting assessments are not routinely used in pediatric practice, (O’Connell et al., 2015) likely due to barriers such as time constraints, lack of reimbursement, lack of validated screening tools, and unproven feasibility.(Fleckman et al., 2021; Smith et al., 2020) One promising exception is the Quick Parenting Assessment (QPA), a validated 13-item parenting assessment and support tool (Sausen et al., 2022). The QPA is designed to support parents, not to identify “bad” parents. Importantly, under no circumstances does the QPA diagnose child abuse (Sausen et al., 2022). To our knowledge, the QPA is the first validated parenting assessment tool to be integrated into the well child visit schedule in at least one clinic (“The Quick Parenting Assessment”, 2020; Sausen et al., 2022).

However, a screening tool that has been validated does not confirm feasibility to scale up. It is unclear if routinely administered parenting assessment tools work in practice. In this context, we conducted an implementation study to assess (1) providers’ time needed to review the QPA and (2) their perceptions about how well it works. The QPA was administered to the parents of children presenting for well visits at specific ages. Then, for these visits, we invited health care providers to complete a survey (i.e. convenience sampling) focused on time needed to review the QPA and their perspectives about the integration process.

## Description

### Design and Setting

This descriptive study was conducted in the Vanderbilt Pediatric Primary Care Clinic in Nashville, Tennessee. The clinic is staffed by 84 pediatric residents, 9 nurse practitioners, and 15 faculty attendings. Families are racially and ethnically diverse: self-identified race are 29% Black/African American, 24% White, 28% Hispanic, and 19% Other or Unknown. Insurance is primarily covered by state government programs with 84% of patients on Medicaid; 12% have private insurance and 3% are uninsured. The IRB at our institution approved the study as a quality improvement project, including implied consent.

In this study, we assessed clinicians’ time and perspectives related to the integration of the QPA, a 13 item questionnaire that assesses for healthy and unhealthy parenting behaviors. The instrument is designed to be educational, evidence-based, brief, never diagnose child abuse, and measure the parenting behaviors of other caregivers (e.g. fathers) who may not attend a clinic visit (Sausen et al., 2022). Appendix 1 lists the QPA items and scoring instructions. Risk groups were determined from a validation study in which, for children ages 4–10, there was twofold increased odds of childhood behavior problems for QPAs of 3–4 and a ninefold increased odds of behavior problems for QPAs > 4, compared to QPAs of 0–2 (Sausen et al., 2022).

In July of 2020, the QPA was integrated into the well child visit by including the instrument in the clinic’s intake forms for parents of 15-month-old children to complete before they saw their health care provider. In March 2021, QPA administration was expanded to include the parents of all children ages 15 months, 30 months, 5 years, and 8 years. Health care providers were trained on how to respond to the QPA with a 10-min presentation during a conference.

### Participants and Survey

Participants were a convenience sample of health care providers who cared for children in the clinic. From September 2020 through March 2022, a QPA Clinician Survey, to be completed anonymously by the provider, was included in the clinic paperwork whenever a parent was given a QPA form to complete. The focus of the study, and the analysis, was on completed, non-excluded surveys, recognizing that some providers may have completed more than one survey. Surveys were eligible to be included in the final data analysis if the parent completed the QPA, the provider reviewed the QPA, and the provider responded to at least one key outcome. Of the 538 surveys that were submitted by providers, 223 were excluded, leaving 315 surveys in the final analysis. Surveys were excluded for at least one (i.e. will not sum to 223) of the following reasons:

1. QPA questions 1–7 were not completed. (N = 59)
2. The provider did not respond to at least 1 important perception outcome question. (N = 130)
3. The provider did not review the QPA with the parent or caregiver. (N = 148)
4. Parent responded “No” to all QPA questions, indicating that a parent may not have read the questions. (N = 63)

## Measures

### Pediatric Health Care Provider Measures

#### The QPA Clinician Survey

The primary measure was the clinician survey, completed by the pediatric provider. It included questions about the provider’s years of experience, provider type (i.e. resident, nurse practitioner, or faculty), and number of previous QPA reviews. Key measures were health care providers’ response to the following:

1. How long did it take to review the QPA with parent?
2. Did the QPA increase your objectivity (i.e. make fewer assumptions) in determining the level of support needed for the caregivers? [Note: we hypothesized increased objectivity given the validation evidence (Sausen et al., 2022)].
3. Did the QPA affect your communications with the caregiver about parenting?
4. Did the QPA affect the quality of the well visit?
5. Did the QPA add value to the visit?
6. Was the parent receptive to the QPA review?
7. Overall, did the QPA work for the visit?

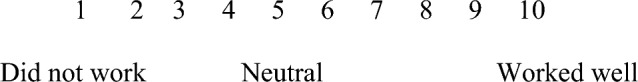


## Parent Measures

### QPA

A secondary measure was the QPA, completed by parents as part of the well child visit at the designated well visit ages listed above.

### Analysis

Survey data was entered into REDCap, a secure online database, and exported into SPSS. We calculated the frequency distribution for the time needed for health care providers to review the QPA, separating the data for low (0–2) and elevated QPAs (> 2). We combined time data for medium risk QPAs (3–4) and high risk QPAs (> 4) because the number of high risk QPAs was relatively small (N = 21) and the distribution of times for medium and high risk QPAs was similar. We calculated the percentages of different QPA levels (low, medium, and high) and responses for the seven key outcomes focused on providers’ perceptions. For the outcome, “Overall, did the QPA work for the visit?”, we grouped the data into 3 categories: “Worked” (> = 7), “Neutral” (4–6), and “Did Not Work” (< = 3). Chi-squared tests were used to assess for associations between providers’ perspectives and QPA scores, provider type, and experience.

## Assessment

### Parent Results

Most QPAs (78.7%) were low risk (QPA <  = 2), 14.6% were medium risk (QPA = 3–4), and 6.7% were high risk (QPA > 4).

### Provider Results

41% of survey forms were completed by residents, 31.4% by nurse practitioners, and 27.6% by attending faculty.

### Time to Review QPA with a Parent

**Fig. 1:**
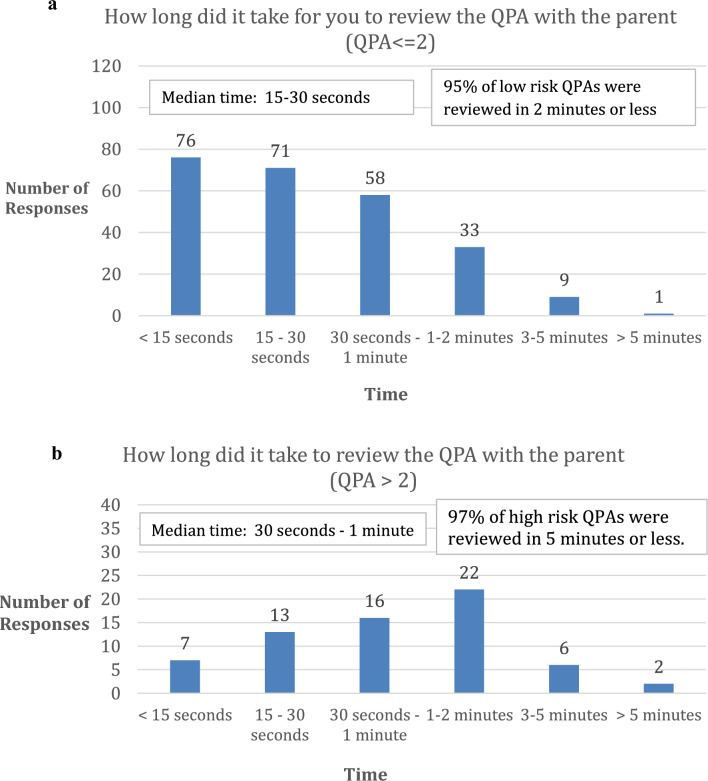
**a** Health care providers’ report of time needed to review low QPAs. **b**. Health care providers’ report of time needed to review elevated QPAs

Health care providers needed more time to review elevated QPAs. Most (95%) low risk QPAs were reviewed in 2 min or less and most (97%) of high risk QPAs were reviewed in 5 min or less (Fig. 1a, b). The median time to review low risk QPAs was 15–30 s and the median time to review elevated QPAs was 30 s to 1 min.

### Health Care Providers’ Perceptions of QPA

**Fig. 2:**
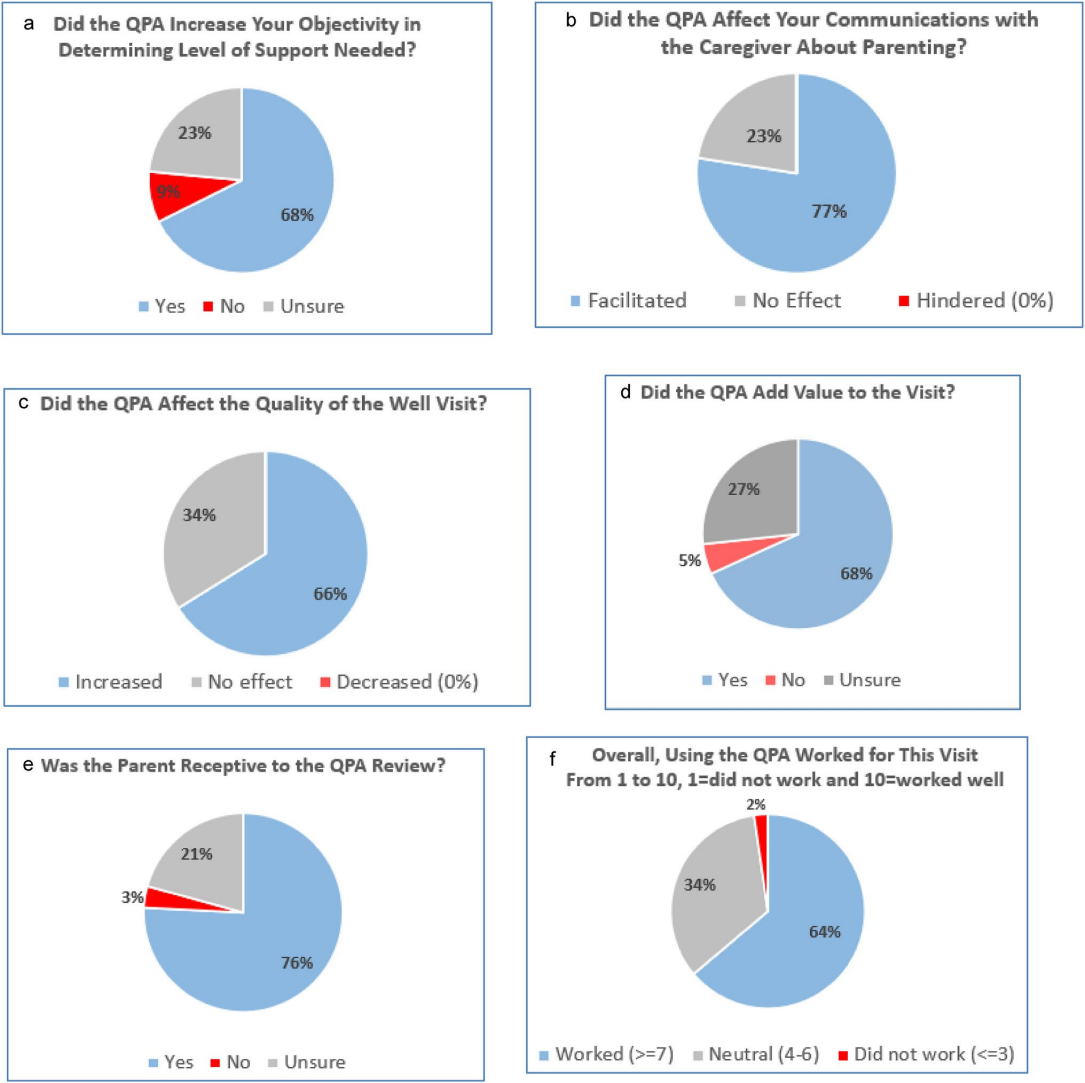
**a**–**f** Health care providers’ key outcome measures

Figure 2a–f illustrate health care providers’ responses to the key measures. For most QPA reviews, health care providers reported that the QPA increased their objectivity in determining the level of support needed for the parent (68%), facilitated communication about parenting (77%), increased the quality of the visit (66%), and increased the value of the visit (68%). Most provider reviews (76%) indicated that the parent was receptive to the QPA review and 3% noted that the parent was not receptive (Fig. 2e). The QPA worked for the visit most of the time (64%) and did not work 2% of the time. No providers reported that the QPA review hindered communication about discipline (Fig. 2b) or decreased the quality of the visit (Fig. 2c).

Health care providers’ responses were categorized based upon the QPA score, their position, experience, age of the child, and number of previous QPA reviews (Table 1). Attendings were more likely to report that the QPA review was beneficial compared to nurse practitioners and residents.

**Table 1:** Providers’ report of their perceptions of reviewing the QPA based upon the QPA score, provider type, experience, age of child, and number of QPA reviews

	The QPA increased objectivity (N = 315)		The QPA facilitated communication (N = 311)		The QPA increased the quality of visit (N = 310)		QPA added value to the visit (N = 311)		The parent is receptive to QPA (N = 314)		The QPA worked overall (N = 307)	
	% yes	p-value	% yes	p-value	% yes	p-value	% yes	p-value	% yes	p-value	% > / = 7	p-value
QPA Score		.667		.360		.568		.568		.523		.618
0–2 (N = 248)	65.7		76.1		64.1		66.8		75.1		62.7	
3–4 (N = 45)	73.3		76.1		68.2		72.1		82.2		69.8	
> 4 (N = 21)	66.7		90.0		75.0		76.1		71.4		68.4	
Provider type		.003*		.044*		.001*		< .001*		.500		< .001*
Attending (N = 86)	80.2		86.0		81.4		86.9		80.2		86.7	
NP (N = 100)	68.0		78.8		66.3		60.0		73.0		49.4	
Resident (N = 126)	57.9		71.5		56.1		62.4		77.2		59.7	
Years of Experience		.326		.940		.103		.980		.0842		.492
< 10 years (N = 218)	65.6		78.0		63.6		68.7		74.0		62.6	
< 10 years (N = 94)	71.3		77.0		73.1		68.8		83.0		66.6	
Age of child		.535		.585		.717		.317		.035*		.870
< = 30 months (N = 265)	71.4		73.4		67.3		73.5		63.5		63.3	
> 30 months (N = 49)	66.9		77.1		64.7		66.2		77.4		58.6	
Number of QPA reviews		.014*		.264		.001*		.102		.627		.126
1–5 times (N = 192)	62.5		74.4		57.8		63.5		68.2		58.9	
> 5 times (N = 120)	75.8		80.0		76.7		72.5		70.8		67.5	

NP indicates Nurse Practitioner

*p value < 0.05

## Discussion

There are many validated parenting assessment tools but, with the exception of the QPA, we are not aware of any that have been integrated into pediatric clinical practice (“The Quick Parenting Assessment”, 2020; Hurley et al., 2014; O’Connell et al., 2015; Sausen et al., 2022). Providers report that the QPA can be reviewed in less than a minute or two, increases communication about discipline, increases their objectivity to offer the appropriate level of support, and adds value to the visit. Pediatric health care providers are well-positioned to support parents in their use of healthy discipline (Taylor et al., 2013) but barriers exist (Fleckman et al., 2021)—these data may lower them.

The most noteworthy barrier to addressing parenting in primary care, reported by 90% of pediatricians, is time constraints (Fleckman et al., 2021). To respond to unhealthy parenting identified through any means (e.g. QPA, behavior problems), health care providers have options. One option is to discuss healthy discipline options in the exam room, realizing that these discussions can become lengthy and, occasionally, emotionally charged. Providers can refer to a parenting intervention—dozens have been demonstrated to work, but parents must attend for months, resulting in participation bias and high attrition, and, in general, there has been inadequate reporting of these programs’ implementation factors (Jeong et al., 2021; Smith et al., 2020). Another option is brief clinical interventions; although they are less potent, brief interventions have potential to reach more parents. One example, with dozens of studies demonstrating effectiveness, is the Play Nicely program (“ Play Nicely: The Healthy Discipline Program”, 2015). Appendix 2 describes how, in one minute, any professional can introduce parents to this evidence-based parenting program that affects parenting and reaches other caregivers who may not attend a clinic visit (e.g. fathers) (Scholer et al., 2024). Finally, assuming the use of electronic health records, smart phrases can, in seconds, be added to a child’s discharge instructions with imbedded links to other vetted programs and resources (Appendix 3). A takeaway message is that there are ways to respond to QPAs, even elevated ones, in minutes, lowering a crucial implementation hurdle.

For providers, time is a driver of costs. Given the link between parenting behaviors, child abuse, and ACEs (see Purpose), this study’s time data should be viewed in the context of the expensive burden of ACEs, and the time needed to review other screening tools that are routinely administered in practice. In the U.S. in 2019–2020, it was estimated that that economic burden of ACEs was $88,000 per affected adult annually (Peterson et al., 2023). Child abuse alone costs hundreds of billions of dollars each year (Fang et al., 2012; Peterson et al., 2018). As for the time/costs of other routine screening practices, Dobrez and colleagues reported on the costs of developmental screening in pediatrics, assuming a 5-min consultation for normal results and 7-to 15-min consultation for abnormal results on a developmental screen (Dobrez et al., 2001). In contrast, we found that most QPAs can be reviewed in less than a minute or two. Future studies could examine the cost-effectiveness of integrating parenting assessment instruments into the well child visit as such work could improve value-based care.

After time constraints, the next most reported barrier to address parenting is reimbursement (Fleckman et al., 2021). Typically, pediatricians receive reimbursement for providing a bundle of primary care services. Pediatricians could substitute one piece of anticipatory guidance with parenting support, resulting in the QPA adding little, if any, additional cost. Additional reimbursement may be approved by payers to implement validated screening tools. To incentivize pediatricians to screen for parenting behaviors, payers could reimburse pediatricians through CPT code 96160, a patient-focused health risk assessment.

Other barriers to educating parents about discipline are not knowing how to counsel parents, concerns about cultural sensitivity, lack of resources, lack of knowledge, lack of confidence, and lack of evidence that counseling is effective (Fleckman et al., 2021). In our clinic, we have made strides to overcome these barriers by (1) developing a 20 min online module to teach why and how to use the QPA (“The Quick Parenting Assessment”, 2020) and (2) using a culturally-sensitive, evidence-based parenting intervention that can be introduced in 1 min (“Play Nicely: The Healthy Discipline Program”, 2015; Scholer et al., 2024; Smith et al., 2017).

Important limitations are that the QPA was implemented in one clinic site and data were derived from a convenience sample, increasing the risk for participation bias. The results are most applicable to health care providers interested in supporting parents with their disciplinary behaviors and willing to complete a survey. Social desirability bias is also possible. Multi-site studies with higher participation rates should be considered. Parents’ perceptions of the QPA are also needed—of note, promising data have been collected, presented at a national meeting, and are available to view (“The Quick Parenting Assessment”, 2020).

## Conclusions

There are excellent reasons to integrate a parenting assessment into practice, but there are barriers. Recognizing study limitations (e.g. convenience sample, single site), these data provide preliminary evidence that a parenting assessment, integrated into the pediatric primary care visit, could lower barriers to educating parents about healthy discipline. The findings have implications for improving parental support in pediatrics, providing value-based care, and preventing poor outcomes associated with ACEs.

## Supplementary Information

Below is the link to the electronic supplementary material.

Supplementary material 1 (pdf 339 kb)

## Data Availability

NA.

## Code Availability

NA.
