# Supplementary material for: Integrating a Parenting Assessment into Practice: Pediatric Providers’ Time and Perspectives
Source: Matern Child Health J. 2024 Sep 16;28(10):1663–70. doi: 10.1007/s10995-024-03984-6 (PMC11420254; doi:10.1007/s10995-024-03984-6)
Supplement: Supplementary file 1 — Supplementary material 1 (pdf 339 kb) [file 10995_2024_3984_MOESM1_ESM.pdf]

Appendix 1: Quick Parenting Assessment

Parents use many options to discipline their children and no parent does it right 100% of the time. To support you and your child, please let us know what YOU and OTHER CAREGIVERS *do*, not what you *think* you should do.

In the past month, what have YOU done when your child needed to be disciplined?

1. You redirected your child toward a good behavior. For example, you said something like: "Don't bite people, use your teeth to eat food" or "Don't kick the dog, let's go kick a ball".	Yes	No
2. In general, more than once per day, you used time-out or took away privilege.	Yes	No
3. In general, more than once per day, you spoke angrily or raised your voice.	Yes	No
4. You told your child that he/she is going to be spanked, smacked, popped, or slapped.	Yes	No
5. You gave your child a spanking, smack, pop, or slap.	Yes	No
6. You told your child something like "You are a bad boy" or "You are a naughty girl".	Yes	No
7. You spent more time with your child to talk about or show them how to improve behavior.	Yes	No

In the past month, what have your child's OTHER CAREGIVERS done when your child needed to be disciplined? SKIP questions 8-12 if you are the only caregiver.

8. In general, more than once per day, they used time-out or took away privilege.	Yes	No
9. In general, more than once per day, they spoke angrily or raised their voice.	Yes	No
10. They told your child that they are going to be spanked, smacked, popped, or slapped.	Yes	No
11. They gave your child a spanking, smack, pop, or slap.	Yes	No
12. They told your child something like "You are a bad boy" or "You are a naughty girl".	Yes	No

Your health care provider wants to support you.

13. I would like to discuss healthy ways to respond to my child's behavior.	Yes	No

Copyright © 2019 Vanderbilt University

Scoring: Q 1 and 7 should be "Yes". However, score only q 2-6, 8-12; all should be "No".

"I would like to review the form you completed about how you discipline your child. Thank you for filling it out. This form is important because how you discipline can affect your child's behavioral and emotional health."

Low Risk (0-2)

"You are using healthy discipline strategies. Keep it up!"

Medium Risk (3-4)

"Parenting can be challenging and humbling. No parent does it right 100% of the time."

Provide healthy discipline education, first building on strengths (q 1 and 7). A one-minute option is the Play Nicely program.[1]

Consider follow up in 1-3 months

If parent does not return in 1-3 months, repeat QPA at the next well visit.

High Risk (>4)

Similar script as "Medium Risk" except consider a stronger recommendation to follow-up in 1-3 months and include community parenting resources.

If parent does not return in 1-3 months, repeat QPA at the next well visit.

Appendix 2:
One Minute Healthy Discipline Education Intervention[1]

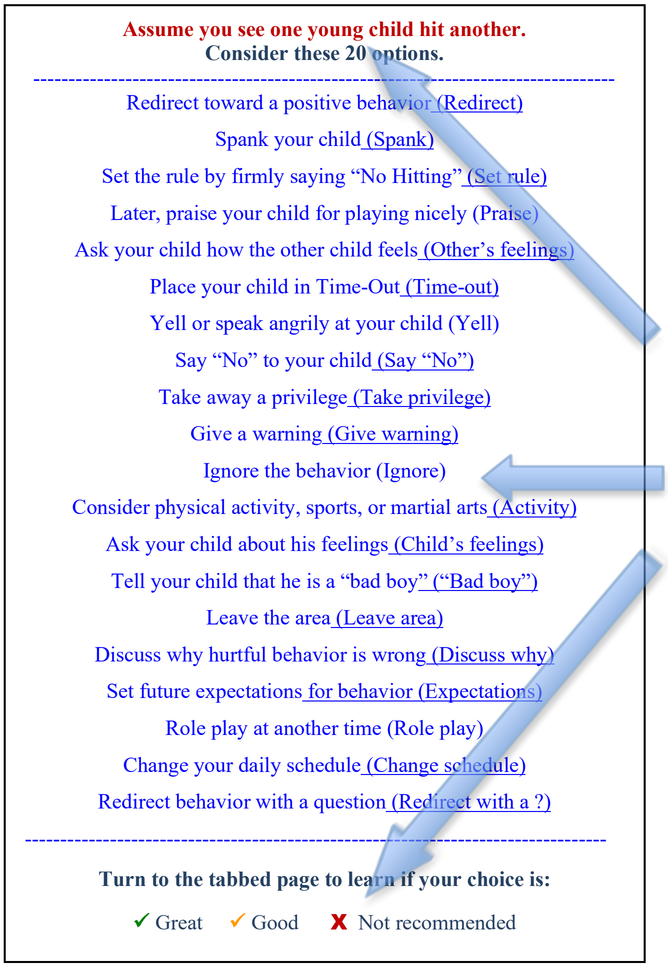

Assume you see one young child hit another.
Consider these 20 options.

Redirect toward a positive behavior (Redirect)
Spank your child (Spank)
Set the rule by firmly saying "No Hitting" (Set rule)
Later, praise your child for playing nicely (Praise)
Ask your child how the other child feels (Other's feelings)
Place your child in Time-Out (Time-out)
Yell or speak angrily at your child (Yell)
Say "No" to your child (Say "No")
Take away a privilege (Take privilege)
Give a warning (Give warning)
Ignore the behavior (Ignore)
Consider physical activity, sports, or martial arts (Activity)
Ask your child about his feelings (Child's feelings)
Tell your child that he is a "bad boy" ("Bad boy")
Leave the area (Leave area)
Discuss why hurtful behavior is wrong (Discuss why)
Set future expectations for behavior (Expectations)
Role play at another time (Role play)
Change your daily schedule (Change schedule)
Redirect behavior with a question (Redirect with a ?)

Turn to the tabbed page to learn if your choice is:

✓ Great ✓ Good ✗ Not recommended

Page 7 of the Play Nicely Healthy Discipline Handbook

Step 1: Introduce 20 Discipline Options

"We want all parents to use healthy discipline strategies with their children. If you haven't already seen it, I would like to introduce you to a great resource called Play Nicely. The program has been proven to increase parents' toolkits for managing behavior problems."

[Turn to page 7 in the Play Nicely Handbook. If the handbook is not available, go to Step 2.]
"Assume you see one young child hit another. What are you going to do?

Here is a list of 20 options."

"Some options are great, some are good after others have been tried, and some are not recommended. Turn to the tabbed pages to learn why redirecting is a great option and why spanking is not recommended. The handbook fills your toolkit with lots of options, so you can pull out one of the better strategies for the situation. The handbook reviews how the options can be modified to use with other childhood behavior problems."

"You can check out the handbook in our Family Resource Center on the 2nd Floor." [Note: if the handbook is not available to check out in your clinic or resource center, parents can purchase the handbook

Step 2: Introduce a Media Option (available in English and Spanish)
"If you would rather learn discipline options by watching a video, you can view a multimedia program on your computer, tablet, or smart phone by going to the website, www.playnicely.org."

Step 3: Get All Parents on Board
"Consider showing the program to everyone who takes care of your child. Your child will behave better if everyone is consistent with discipline."

Step 4: Follow up
"If you have any ongoing concerns about your child's behavior or about how to discipline your child, please come back and see me in 1-3 months."

1. Scholer SJ, Martin HK, Adams L, Dietrich MS. A Brief Intervention in Primary Care to Improve Parents' Discipline Practices and Reach Other Caregivers. *Clinical pediatrics.* 2024:99228241227756.

Appendix 3:
Quick Parenting Assessment (QPA)
Discharge Instructions

Health care providers usually give discharge instructions to patients. At our institution, our electronic health record, Epic, uses Smart Phrases that can add discharge instructions in seconds (e.g. typing ".QPALOW"). Below are examples of text that we use to respond to low, medium, and high QPAs.

.QPALOW: Healthy Discipline: Our goal is for your child to have excellent emotional and behavioral health. A first step is for parents to use healthy discipline strategies. You are doing it! Keep it up!

Healthy discipline resources
www.playnicely.org
www.cdc.gov/parents
www.zerotothree.org

.QPAMEDIUM: Healthy Discipline: We want to support you to help your child have excellent emotional and behavioral health. A first step is for parents to use healthy discipline strategies. Your child will likely have better behavioral health if you and your child's other caregivers use the best discipline strategies to respond to behavior problems.

Although there is no one right way to respond to behavior problems, some discipline strategies work better than others. Try to avoid spanking, yelling, or threatening or saying "No" too much because these do not teach children what they are supposed to do, and these strategies can be unhealthy for your child. First, take the time to explain the rules, using good eye contact. Then, one of the best discipline strategies is to redirect a child toward a more positive behavior. For example, if your child hits another, redirect your child to go build blocks, throw a ball, or help you around the house. If your child kicks, have them go kick a soccer ball or run up and down the hall with their feet. Redirecting works great because you give your child attention, and at the same time, you teach them what they can do, not what they can't.

To learn about healthy ways to discipline, please view the Play Nicely multimedia program at home on your computer or smart phone (see www.playnicely.org). You can check out the Play Nicely Healthy Discipline Handbook from the Family Resource Center on the 2nd Floor of the Doctor's Office Tower. Show the program to everyone who takes care of your child. Your child will behave better if everyone is consistent with discipline. Please use the strategies in the program and come back to see me in 1-3 months, especially if you have any questions about healthy discipline options or your child's behavior.

Healthy discipline resources
www.playnicely.org
www.cdc.gov/parents
www.zerotothree.org

.QPAHIGH: Same as .QPAMEDIUM except 1) Please use the strategies in the program and come back to see me in 1-3 months and 2) include a list of community parenting classes and support groups.
